# Graft-Versus-Leukemia Effect in the Management of Isolated Pancreatic Granulocytic Sarcoma Following Allogeneic Stem Cell Transplantation

**DOI:** 10.7759/cureus.90072

**Published:** 2025-08-14

**Authors:** Ramazan Kurt, Yucel Aydin, Sevgi Kalayoglu Besisik

**Affiliations:** 1 Internal Medicine, Istanbul University School of Medicine, Istanbul, TUR; 2 Internal Medicine/Hematology, Istanbul University Istanbul Medical Faculty, Istanbul, TUR

**Keywords:** allogeneic stem cell transplantation, chloroma, extramedullary aml, graft-versus-leukemia effect, granulocytic sarcoma, myeloid sarcoma, pancreas, preleukemic gs

## Abstract

Granulocytic sarcoma (GS), also known as chloroma, is a rare extramedullary tumor composed of immature myeloid cells, most often seen in association with acute myeloid leukemia (AML). Isolated GS without bone marrow involvement is extremely uncommon, and fewer than 15 cases of isolated pancreatic GS have been reported in the literature. It is frequently misdiagnosed due to its morphological resemblance to lymphoid malignancies. We present the case of a 19-year-old male who developed obstructive jaundice and was found to have a pancreatic mass initially interpreted as a peripheral T-cell lymphoma. Further biopsy and immunohistochemical analysis confirmed the diagnosis of GS, with strong positivity for myeloperoxidase, lysozyme, and CD68. Bone marrow evaluation and cytogenetic analysis were normal, confirming isolated disease. The patient was treated with AML-based chemotherapy, which resulted in only a minimal reduction in tumor size. Nine months later, he underwent allogeneic stem cell transplantation (allo-SCT) from an HLA-identical sibling. Follow-up imaging at four months post-transplant revealed a dramatic reduction of over 90% in the mass size. At eight months, the patient remained in complete clinical and hematologic remission. This case highlights the diagnostic and therapeutic challenges of isolated GS in an atypical site and provides evidence for a potential graft-versus-leukemia effect in extramedullary disease. The post-transplant tumor regression, despite limited chemotherapy response, underscores the significance of immune-mediated control and suggests a possible role for allo-SCT even in preleukemic GS.

## Introduction

Granulocytic sarcoma (GS), also known as chloroma or myeloblastoma, is a rare extramedullary tumor composed of immature myeloid cells [1]. It most often occurs in association with acute myeloid leukemia (AML) but may also arise during myelodysplastic or myeloproliferative disorders [1]. In some cases, GS appears without bone marrow involvement, termed preleukemic GS, in contrast to leukemic GS, which is accompanied by systemic disease [2,3]. Preleukemic GS often precedes the development of AML within months if untreated and requires prompt systemic therapy [4].

Diagnosis can be challenging, as GS frequently mimics lymphoid malignancies on morphology and imaging [3]. Immunohistochemical staining for myeloid markers such as myeloperoxidase (MPO), lysozyme, and CD68 is critical for confirmation [2,4]. Histological distinction from high-grade lymphoma is essential, as treatment strategies differ markedly [5]. Pancreatic involvement is extremely rare; fewer than 15 cases of isolated pancreatic GS have been reported in the literature, most in the context of established AML, making this an exceptional presentation [6].

There is no standardized treatment, but AML-based chemotherapy regimens have been shown to delay or prevent leukemic transformation more effectively than local therapies alone [7,8]. Allogeneic stem cell transplantation (allo-SCT) is a potentially curative option, particularly in high-risk or refractory disease [8,9]. Beyond the cytotoxic effects of conditioning, allo-SCT provides an immune-mediated graft-versus-leukemia (GVL) effect, in which donor immune cells target residual malignant cells [9]. While the GVL effect is well recognized in marrow disease, its role in preleukemic or extramedullary GS remains less clear. Limited reports describe regression of extramedullary GS following donor lymphocyte infusion or non-myeloablative allo-SCT [10-12].

Here, we report a rare case of isolated pancreatic GS presenting with obstructive jaundice, initially misdiagnosed as peripheral T-cell lymphoma. The lesion showed minimal response to AML-based chemotherapy but underwent marked regression following allo-SCT, suggesting that a post-transplant GVL effect, independent of chemotherapy, may be capable of eradicating preleukemic GS in rare extramedullary sites.

## Case presentation

A 19-year-old previously healthy male presented with a one-month history of progressive fatigue, unintentional weight loss, scleral icterus, dark urine, and pale stools. He denied fever, abdominal pain, or pruritus. On examination, he was pale and jaundiced, with a well-healed surgical scar in the right upper quadrant from a prior cholecystectomy. No lymphadenopathy or hepatosplenomegaly was detected.

Laboratory evaluation revealed total bilirubin of 12.7 mg/dL (direct: 9.8 mg/dL), elevated alkaline phosphatase (780 U/L) and gamma-glutamyl transferase (465 U/L), and mildly increased transaminases. Viral hepatitis serologies were negative. Abdominal ultrasonography showed dilated intra- and extrahepatic bile ducts, an enlarged gallbladder, and a hypoechoic mass compressing the distal common bile duct, with peripancreatic and para-aortic lymphadenopathy.

The patient initially underwent exploratory laparotomy with choledochoduodenostomy for biliary decompression and fine-needle aspiration biopsy (FNAB) of the pancreatic head mass at the referring hospital. FNAB, performed prior to cross-sectional imaging, suggested infiltration by atypical peripheral T lymphocytes, raising concern for lymphoma. Immunohistochemistry could not be completed due to insufficient material, and the patient was referred to our center for further evaluation.

**Figure 1:**
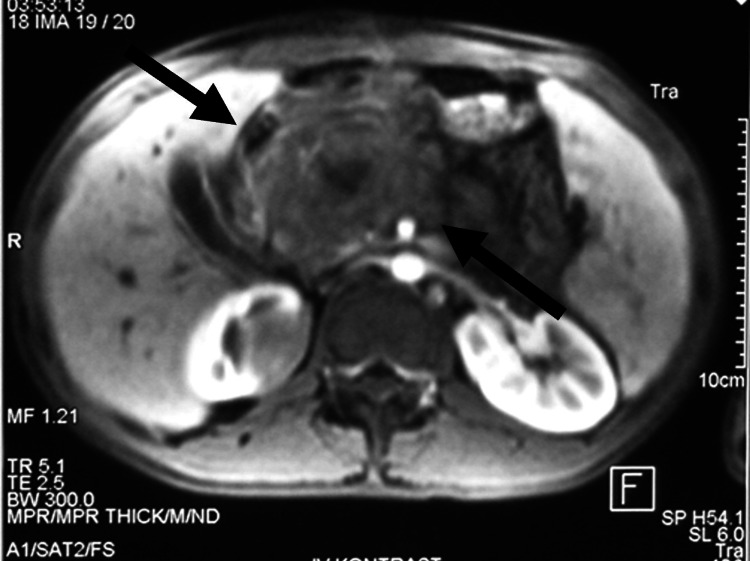
Contrast-enhanced abdominal magnetic resonance imaging showing a 5.6 × 4.2 cm heterogeneous mass (arrow) in the head of the pancreas with associated biliary dilatation and regional lymphadenopathy. A smaller lesion is visible near the upper pole of the right kidney.

At our institution, contrast-enhanced MRI revealed a 5.6 × 4.2 cm heterogeneous mass in the pancreatic head with regional lymphadenopathy and a smaller lesion near the upper pole of the right kidney (Figure 1).

**Figure 2:**
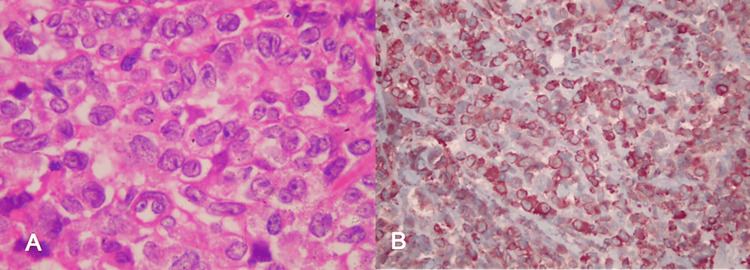
Histopathologic and immunohistochemical findings of the pancreatic mass. (A) Hematoxylin and eosin stain showing diffuse infiltration of medium-to-large mononuclear cells with high nuclear-to-cytoplasmic ratio and prominent nucleoli (original magnification ×400). (B) Strong cytoplasmic positivity for myeloperoxidase (original magnification ×100).

The differential diagnoses included high-grade lymphoma, pancreatic adenocarcinoma, autoimmune pancreatitis, and, less likely, granulocytic sarcoma. A percutaneous Tru-Cut core needle biopsy was performed. Histology demonstrated diffuse infiltration by medium-to-large mononuclear cells with a high nuclear-to-cytoplasmic ratio, vesicular chromatin, and prominent nucleoli. Immunohistochemical staining showed strong positivity for myeloperoxidase (MPO), lysozyme, and CD68, with weak scattered staining for CD3, CD5, and CD20, confirming granulocytic sarcoma (Figure 2).

Bone marrow aspirate and trephine biopsy showed normocellular marrow with trilineage hematopoiesis and no leukemic infiltration. Cytogenetics and FISH for common AML-associated abnormalities (t(8;21), inv(16), monosomy 7) were negative. Next-generation sequencing (FLT3, NPM1, CEBPA) was unavailable due to technical limitations at the institution at that time.

Given the aggressive nature of GS, the patient was started on standard AML induction chemotherapy with the “7 + 3” regimen (continuous cytarabine for seven days plus daunorubicin on days 1-3), followed by two cycles of high-dose cytarabine consolidation. Imaging after induction and consolidation showed only minimal reduction in tumor size, despite complete hematologic recovery and no marrow progression.

Nine months after diagnosis, he underwent allogeneic stem cell transplantation (allo-SCT) from his human leukocyte antigen (HLA)-identical sibling donor. Myeloablative conditioning included busulfan and cyclophosphamide, followed by infusion of 3.88 × 10⁶/kg CD34⁺ cells. Graft-versus-host disease (GVHD) prophylaxis consisted of cyclosporin A and methotrexate. Neutrophil engraftment occurred by day +22 without major complications.

**Figure 3:**
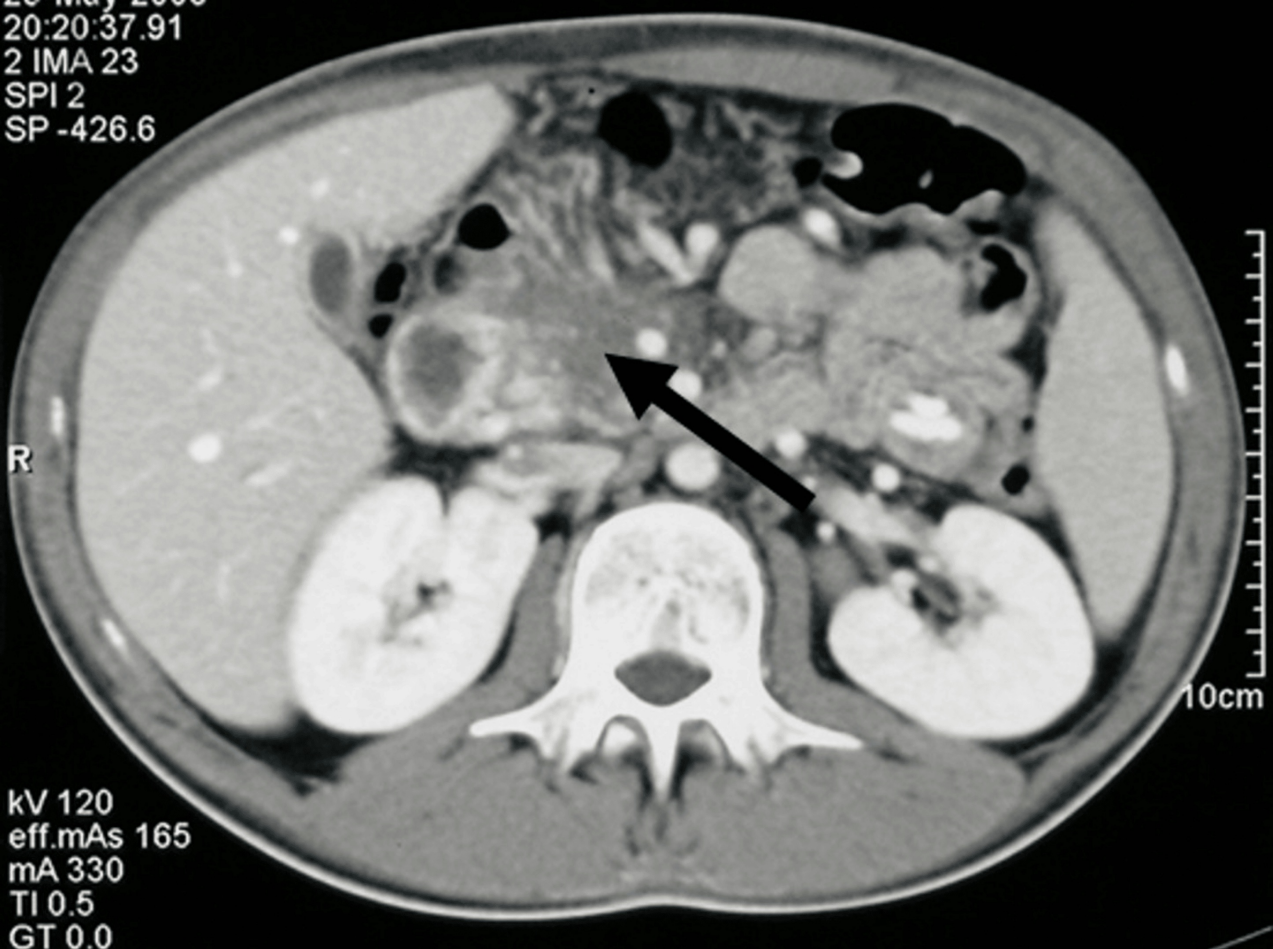
Abdominal computed tomography scan four months after allogeneic stem cell transplantation, demonstrating >90% reduction in pancreatic tumor size (arrow), consistent with a possible graft-versus-leukemia effect.

Post-transplant CT at one month showed a persistent pancreatic mass. By the fourth month, imaging revealed a >90% reduction in tumor size (Figure 3).

A concurrent bone marrow biopsy confirmed complete donor chimerism without leukemic transformation. At eight months post-transplant, the patient remained asymptomatic, with normal laboratory values and no radiologic evidence of disease. He remains in complete remission and is undergoing planned long-term surveillance with annual periodic positron emission tomography-computed tomography (PET-CT) scans.

## Discussion

GS, also referred to as chloroma or myeloid sarcoma, is an extramedullary tumor composed of immature myeloid cells. While most cases are associated with AML, GS can also occur in a preleukemic phase, precede systemic disease, or present at relapse. Isolated GS without bone marrow involvement is rare and presents significant diagnostic challenges. Morphologic and radiologic similarities to lymphoid neoplasms or solid tumors frequently lead to misdiagnosis, as initially occurred in our patient. Immunohistochemistry (IHC) is essential for accurate diagnosis, with MPO, lysozyme, and CD68 confirming myeloid lineage and excluding mimickers such as high-grade non-Hodgkin lymphomas [1,2]. In our case, a deep percutaneous core biopsy allowed adequate IHC. Markers for lymphoid neoplasms (CD3, CD5, and CD20), cyclin D1, and BCL2 were negative, and EBER in situ hybridization was also negative, effectively excluding lymphoid and viral-driven differentials.

Common GS sites include skin, bone, lymph nodes, orbits, and central nervous system (CNS) [3]. Pancreatic involvement is extremely rare, with only a handful of reported cases, most in the setting of established AML [4]. Among published cases, isolated pancreatic GS without marrow disease is exceptional, further underscoring this report’s novelty.

The natural course of untreated or locally treated GS is poor, with progression to AML occurring in most cases within months [5,6]. For this reason, AML-type systemic chemotherapy is recommended even in isolated GS [6,7]. Our patient received standard induction (7+3) followed by two cycles of HiDAC consolidation. Tumor size decreased only minimally, indicating chemoresistance despite normal marrow and no leukemic transformation.

Allo-SCT is potentially curative for high-risk AML and GS, offering both cytotoxic effects from conditioning and the immune-mediated GVL effect [8,9]. GVL is mediated by donor-derived T lymphocytes recognizing leukemia-associated or minor histocompatibility antigens [9]. While GVL is well established in systemic AML, its role in isolated extramedullary disease is less defined.

In our patient, allo-SCT was performed after a suboptimal chemotherapy response. Extramedullary GS may demonstrate chemoresistance due to factors such as poor drug penetration into the tumor (sanctuary site effect) and the influence of the tumor microenvironment on drug resistance. Conditioning included myeloablative busulfan and cyclophosphamide. Early post-transplant CT (day +30) showed stable tumor size, suggesting limited cytoreduction from conditioning alone. By month four, imaging demonstrated >90% reduction, and complete donor chimerism was documented. This delayed but profound regression strongly supports a post-transplant immune-mediated effect. While we attribute this to GVL, other possibilities include delayed cytotoxicity or spontaneous regression, although the latter is exceedingly rare. Similar delayed responses have been reported in extramedullary GS after allo-SCT or DLI [10-12].

Notably, our patient did not develop GVHD, suggesting that GVL can occur independently. Although GVHD and GVL often overlap, selective GVL without GVHD is possible, potentially via preferential targeting of leukemia-specific antigens.

This is among the very few reports describing dramatic regression of isolated pancreatic GS after allo-SCT in a preleukemic state. Given the high risk of progression and chemoresistance in such cases, early transplant consideration should be made, particularly in young, fit patients or those with high-risk anatomical sites.

Further studies are needed to define the optimal timing of allo-SCT, the role of DLI, and the durability of GVL in extramedullary GS. This case reinforces that GVL may extend beyond marrow disease, offering curative potential in rare and diagnostic challenging forms of myeloid sarcoma.

## Conclusions

Isolated GS of the pancreas is an exceptionally rare and diagnostically challenging presentation, often mimicking lymphoid malignancies or solid tumors. Accurate diagnosis requires adequate tissue sampling and IHC to confirm myeloid lineage and exclude mimickers. While AML-based chemotherapy remains the cornerstone of initial management, chemoresistance in extramedullary disease is not uncommon.

This case demonstrates that allo-SCT can achieve profound and durable remission in isolated GS, likely through a delayed but potent GVL effect. Importantly, GVL may occur in the absence of GVHD, underscoring its potential as a targeted immunologic mechanism. Early consideration of allo-SCT should be made in young, fit patients with isolated GS, especially when chemotherapy response is suboptimal or the disease involves high-risk anatomical sites. Further investigation is warranted to better define the role, timing, and long-term outcomes of allo-SCT in isolated extramedullary GS, as well as strategies to harness GVL while minimizing GVHD.
